# Dual pathology and Takotsubo syndrome. A case report

**DOI:** 10.1192/j.eurpsy.2025.1000

**Published:** 2025-08-26

**Authors:** M. Valverde Barea, A. Jurado Arevalo, I. Contreras Pérez, M. P. Vargas Melero

**Affiliations:** 1 H. U. Jaén, Jaén, Spain

## Abstract

**Introduction:**

Cocaine use is a significant health problem, since its abuse constitutes a substance use disorder and can produce cardiovascular symptoms, including acute coronary syndrome (ACS). Tako-tsubo syndrome (TTS) is a transient dysfunction of the apical left ventricle that can manifest with intense chest pain and alterations in the electrocardiogram. TTS can be triggered by physical stressors (hyperthyroidism, cocaine or amphetamine abuse) or psychological stressors (grief or reaction to acute stress).

**Objectives:**

The objective is to present the case of a patient who developed tako-tsubo syndrome after harmful cocaine use.

**Methods:**

A 50-year-old woman, under psychiatric follow-up, diagnosed with mixed personality disorder, with a medical history of hypothyroidism. The patient has been presenting for 2 months after mourning the death of her husband with anxiety-depressive symptoms with a great behavioral impact with episodes of heteroaggression and begins with abusive consumption of cannabis and cocaine toxins. The patient presents an episode of psychomotor agitation after acute consumption of cocaine, presenting psychotic symptoms. During the study in the emergency area, she verbalizes chest pain and complementary studies are performed with elevated cardiac enzymes and alterations in the electrocardiogram. Cardiology is contacted and after hospital admission and complementary studies, the diagnosis of acute coronary syndrome is reached, filiating it with Takotsubo syndrome triggered by cocaine consumption. The diagnoses presented by the patient with dual pathology would be a psychotic disorder due to toxins, a borderline personality disorder and harmful consumption of cocaine.

**Results:**

Dual pathology in patients with personality disorder with toxin abuse such as in this case cocaine is very frequent.The situation of grief is an acute stressful situation that leads to greater emotional instability and, together with the toxic effects of cocaine, can trigger Takotsubo syndrome in patients. Therefore, when approaching patients with substance use, it is very important to always take into account the possible somatic complications that can be triggered.The treatment chosen to treat the psychotic symptoms that the patient presented was lurasidone, since it has a tolerability and cardiological safety profile.

**Conclusions:**

In patients with dual pathology, it is very important to address all possible complications, both somatic and psychiatric, for proper therapeutic management.

**Disclosure of Interest:**

None Declared

